# Neutrophil-to-Lymphocyte Ratio and Platelet-to-Lymphocyte Ratio Impact on Predicting Outcomes in Patients with Acute Limb Ischemia

**DOI:** 10.3390/life12060822

**Published:** 2022-05-31

**Authors:** Emil Marian Arbănași, Adrian Vasile Mureșan, Cătălin Mircea Coșarcă, Réka Kaller, Theodor Ioan Bud, Ioan Hosu, Septimiu Toader Voidăzan, Eliza Mihaela Arbănași, Eliza Russu

**Affiliations:** 1 Clinic of Vascular Surgery, Mureș County Emergency Hospital, 540136 Targu-Mures, Romania; emilarbanasi1@gmail.com (E.M.A.); adrian.muresan@umfst.ro (A.V.M.); catalin.cosarca@umfst.ro (C.M.C.); reka.kaller@umfst.ro (R.K.); theodor.bud@gmail.com (T.I.B.); eliza.russu@umfst.ro (E.R.); 2 Department of Surgery, University of Medicine, Pharmacy, Science and Technology “George Emil Palade” of Targu-Mures, 540139 Targu-Mures, Romania; 3 Department of Nephrology, Mureș County Emergency Hospital, 540136 Targu-Mures, Romania; ioan.hosu@umfst.ro; 4 Department of Epidemiology, University of Medicine, Pharmacy, Science and Technology “George Emil Palade” of Targu-Mures, 540139 Targu-Mures, Romania; septimiu.voidazan@umfst.ro; 5 Faculty of Pharmacy, University of Medicine, Pharmacy, Science and Technology “George Emil Palade” of Targu-Mures, 540139 Targu-Mures, Romania

**Keywords:** acute limb ischemia, vascular surgery, embolectomy, mortality, amputation

## Abstract

Acute Limb Ischemia (ALI) of the lower limb is defined as a sudden drop in arterial limb perfusion, which is a medical emergency requiring prompt intervention with high amputation and mortality rates in the absence of revascularization. This observational, analytical, and retrospective cohort study with longitudinal follow-up aimed to confirm the relevance of the preoperative inflammatory biomarkers neutrophil-to-lymphocyte ratio (NLR) and platelet-to-lymphocyte ratio (PLR) in predicting the 30-day poor prognosis of patients with Rutherford classification (RC) grades II and III ALI. The ROC analysis found a strong association of an NLR > 4.33 with all studied outcomes, while a PLR > 143.34 was associated with all studied outcomes, except the composite endpoint in all RC stages. Depending on the optimal cut-off value, the ROC analysis found a higher incidence of all adverse outcomes in all high NLR (>4.33) and high PLR (>143.34) groups. A multivariate analysis showed that a high baseline value for NLR and PLR was an independent predictor of amputation (OR:11.09; 95% CI: 5.48–22.42; *p* < 0.0001; and OR:8.97; 95% CI: 4.44–18.16; *p* < 0.0001), mortality (OR:22.24; 95% CI: 9.61–51.47; *p* < 0.0001; and OR:8.32; 95% CI: 3.90–17.73; *p* < 0.0001), and composite endpoint (OR:21.93; 95% CI: 7.91–60.79; *p* < 0.0001; and OR:9.98; 95% CI: 3.89–25.55; *p* < 0.0001), respectively. Furthermore, for all hospitalized patients, the RC grade III (OR:7.33; 95% CI: 3.73–14.26; *p* < 0.0001) was an independent predictor of amputation (OR:7.33; 95% CI: 3.73–14.26; *p* < 0.0001), mortality (OR:8.40; 95% CI: 4.08–17.31; *p* < 0.0001), and composite endpoint (OR: 10.70; 95% CI: 4.48–25.56; *p* < 0.0001), respectively. The NLR and PLR are excellent predictors of risks associated with ALI for primary and secondary prevention. Our study showed that increased pre-operative values for NLR and PLR are indicators of a poor outcome in patients with RC grades II and III ALI.

## 1. Introduction

Acute Limb Ischemia (ALI) of the lower limb is defined as a sudden drop in arterial limb perfusion, which is clinically manifested by pallor and cold skin, decreased sensitivity and motility, decreased muscle strength, claudication, and the absence of a pulse distal to the site of occlusion [1,2]. It is a medical emergency with an incidence of 3–14 per 100,000 persons/year [1] and requires immediate care as it involves high rates of amputation and mortality in the absence of revascularization [3,4].

The most common causes of ALI are embolism and in situ thrombosis of an atherosclerotic plaque, while other causes such as stent or graft blockage, trauma, or thrombosis of the lower limb artery aneurysms present with a reduced frequency [5,6,7]. The Rutherford classification (RC) determines the severity of ALI with four grades ranging from moderate to severe, starting from nonthreatened, viable limbs (grade I) and progressing to grade III (skin, muscle, and nerve deterioration) [8].

The development of a biomarker with a predictive function in the incidence of postoperative complications in ALI patients is a topic that has lately received a lot of attention. The neutrophil-to-lymphocyte ratio (NLR) is one of the most widely available biomarkers with a significant association with morbidity and mortality in the following study areas: cardiovascular surgery, cardiology, gastric cancer, pancreatic cancer, colorectal cancer, and ovarian cancer [9,10,11,12,13,14,15,16]. Another biomarker of interest is the platelet-to-lymphocyte ratio (PLR), which is intensively studied in the fields of oncology [17,18,19], cardiovascular surgery [20], bipolar disorder [21], and trauma care [22]. The proinflammatory role of neutrophils and lymphocytes in immune system modulation has been widely described in the literature [23,24], as well as the role of systemic inflammation in lymphocyte apoptosis [25], possibly leading to adverse cardiovascular events [26].

The predictive role of the biomarkers in ALI is of great interest, as time is crucial in vascular emergencies, and laboratory findings may help significantly in choosing the best care and performing accurate risk stratification. Unfortunately, there are no precise cut-off values for these biomarkers in ALI clinical guidelines. Furthermore, the most extensively studied biomarker is NLR, without associating the other biomarker in acute scenarios. The most recent papers regarding the predictive role of NLR in ALI are that of Taurino et al. [27], Tasoglu et al. [28], and Pasqui et al. [29], who established that an NLR > 5 NLR was strongly associated with poor outcomes in ALI patients.

Starting from the findings of Taurino et al., Tasoglou et al., and Pasqui et al., this study aimed to verify the predictive role of the preoperative inflammatory biomarkers NLR and PLR in predicting the 30-day poor outcome of patients with RC II and III ALI.

## 2. Materials and Methods

### 2.1. Study Design

The present study was designed as an observational, analytical, and retrospective cohort study with a longitudinal follow-up and included all patients with a diagnosis of ALI admitted to the Vascular Surgery Clinic of the County Emergency Clinical Hospital of Târgu-Mureș, Romania between January 2017 and December 2019. The exclusion criteria were as follows: RC grade I, systemic inflammatory disease, recent tumor status, hematological diseases, personal history of major surgery in the previous six months, and autoimmune diseases.

Patients included in the study were initially divided into two groups depending on their poor outcomes: patients who survived and those who died. Furthermore, we used the receiver operating characteristic (ROC) curve to identify the optimal cut-off values of NLR and PLR for all poor outcomes. The univariate and multivariate analyses were performed to verify the predictive role of the preoperative inflammatory biomarkers NLR and PLR in predicting the 30-day poor outcome of patients with RC II and III ALI.

### 2.2. Data Collection

The patients’ demographic data were extracted from the hospital’s electronic database. We searched for the following comorbidities in the medical history: arterial hypertension (AH), ischemic heart disease (IHD), atrial fibrillation (AF), myocardial infarction (MI), chronic heart failure (CHF), chronic obstructive pulmonary disease (COPD), chronic kidney disease (CKD), type 2 diabetes (T2D), stroke, chronic venous insufficiency (CVI), dyslipidemia, tobacco use, and obesity.

### 2.3. Preoperative Workup and Revascularization Technique

Before the surgery, physical examination, and blood tests (glucose level, hemoglobin, hematocrit, neutrophil count, lymphocyte count, monocyte count, and platelet count) were performed. The NLR and PLR were calculated using the equations below:

$$\mathrm{NLR}=\frac{\mathrm{total}\;\mathrm{number}\;\mathrm{of}\;\mathrm{neutrophils}}{\mathrm{total}\;\mathrm{number}\;\mathrm{of}\;\mathrm{lymphocytes}}$$

$$\mathrm{PLR}=\frac{\mathrm{total}\;\mathrm{number}\;\mathrm{of}\;\mathrm{platelets}}{\mathrm{total}\;\mathrm{number}\;\mathrm{of}\;\mathrm{lymphocytes}}$$

Fogarty embolectomy and bypass were used for revascularization. Major amputations were performed in patients without any indication of revascularization and, in all cases, were performed above the ankle. The therapeutic approach was chosen based on the patient’s general status, level of arterial occlusion, grade of atherosclerotic disease, and the surgeon’s expertise.

### 2.4. Study Outcomes

The primary endpoints were 30-day mortality rate, major amputation rate, and a composite endpoint of amputation and mortality. Outcomes were stratified for RC at hospital admission and for the optimal NLR and PLR cut-off value at baseline.

### 2.5. Statistical Analysis

A statistical analysis was performed using SPSS for Mac OS version 28.0.1.0 (SPSS, Inc., Chicago, IL, USA). For the normal data distribution, the results were expressed as mean ± SD, while for nonparametric distribution, median and interquartile ranges were used. The categorical variables were expressed as number (no.) and percentage (%) of patients from the cohort. The associations of NLR and PLR with category variables were assessed using Chi-square tests, while differences in continuous variables were analyzed using Student’s *t*-tests or Mann–Whitney tests. The receiver operating characteristic (ROC) curve analysis was used to identify the optimal cut-off values of NLR and PLR according to the Youden index (Youden Index = Sensitivity + Specificity − 1, ranging from 0 to 1). A multivariate logistic regression analysis that included variables with *p* < 0.1 was performed to identify independent predictors of amputation, mortality, and a composite endpoint of amputation and mortality. All tests were two-tailed, and a *p*-value < 0.05 was considered statistically significant.

## 3. Results

**Figure 1:**
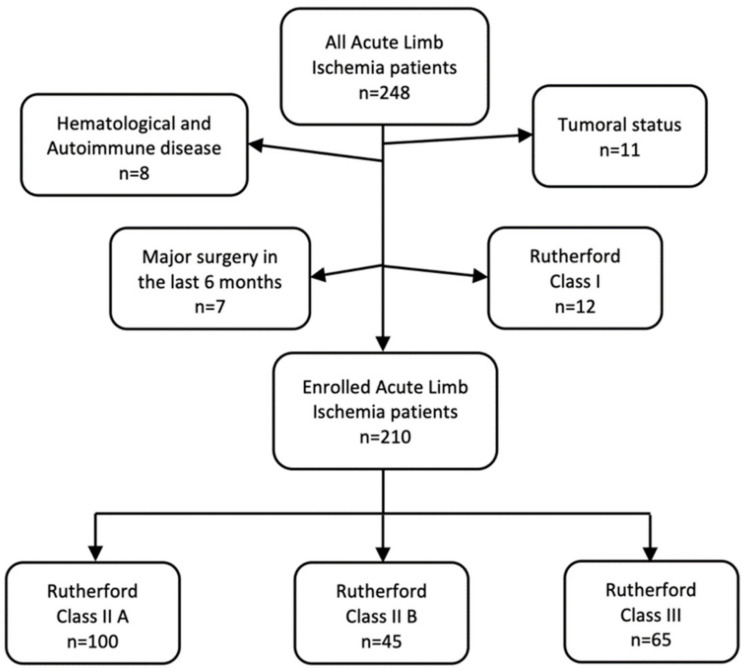
Enrollment flowchart.

During the study period, 248 patients with ALI were admitted. Of these, 11 had tumoral status; 8 had autoimmune or hematological diseases; 7 had major surgery in the previous six months, and 12 were diagnosed with Rutherford grade I arterial disease. As a result, 210 patients were enrolled in the study: 100 with RC IIA, 45 with RC IIB, and 65 with RC III (Figure 1).

One hundred fifty-three patients (72.86%) were male. The mean age of the patients was 69.56 ± 8.21, ranging from 51 to 92 years. The comorbidities with the highest incidence were: AH (83.33%), IHD (80.48%), CHF (60.48%), and T2D (52.86%), while the risk factors present were smoking (63.81%), hyperlipidemia (59.05%), and obesity (33.81%). The rest of the comorbidities and laboratory data are presented in Table 1.

Among the operations performed, Fogarty embolectomy was performed in 145 cases (69.05%), a bypass in 48 cases (22.86%), and major amputation in 17 cases (8.1%). Fifty-seven patients (27.14%) required amputation in the first 30 days, and 47 patients (22.38%) died (Table 1).

Depending on the 30-day mortality, the patients were enrolled in two groups. Patients in the first group had a favorable 30-day result, whereas those in the second group had a negative 30-day outcome. Table 2 show the gender distribution, mean age, comorbidities, laboratory data, Rutherford classification, type of surgery, and amputation rate.

In terms of laboratory findings, the group with poor outcomes had lower hemoglobin values (*p* = 0.0005), as well as lower values for hematocrit (*p* < 0.0001) and lymphocytes (*p* < 0.0001). The poor outcome group also presented high values for glucose (*p* = 0.01), neutrophils (*p* < 0.0001), monocytes (*p* = 0.03), platelets (*p* = 0.02), and NLR (*p* < 0.0001), and PLR (*p* < 0.0001) ratios (Table 2).

Significant differences were found between the two groups in terms of the Rutherford grading: in the positive outcome group, a statistically higher number of patients was admitted with grade IIA (*p* < 0.0001), whereas in the adverse outcome group, there was a higher incidence of grade III (*p* < 0.0001). In terms of the surgery performed, the poor outcome group had more cases of major amputation as a mandatory first procedure (*p* = 0.0006). Furthermore, in the first 30 days, there were 17 patients who needed an amputation in the positive outcome group, compared to 23 in the other group. In 30 days, there was an overall higher amputation rate in the poor outcome group (70.21% vs. 14.72%; *p* < 0.0001) (Table 2).

**Figure 2:**
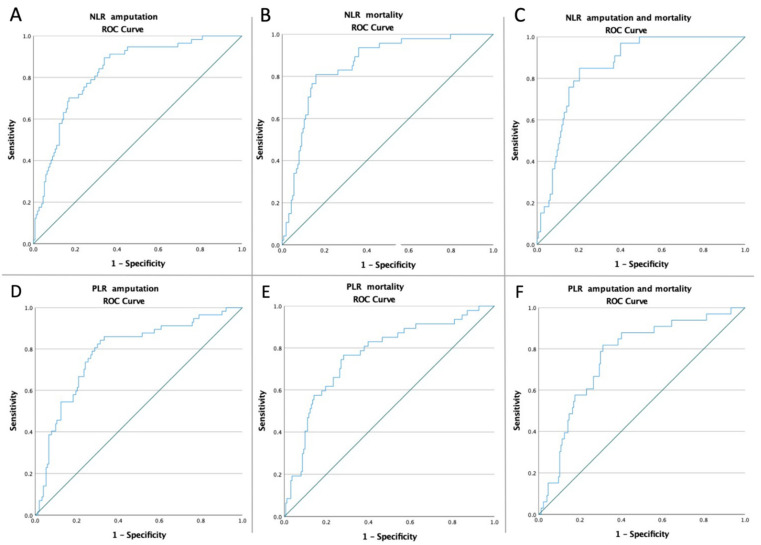
ROC curve analysis (**A**) for NLR concerning amputation rate in all patients (AUC: 0.829), (**B**) for NLR concerning mortality rate in all patients (AUC: 0.856), (**C**) for NLR concerning amputation and mortality rates in all patients (AUC: 0.858), (**D**) for PLR concerning amputation rate in all patients (AUC: 0.785), (**E**) for PLR concerning mortality rate in all patients (AUC: 0.765), (**F**) for PLR concerning amputation and mortality rates in all patients (AUC: 0.759); NLR = neutrophil-to-lymphocyte ratio; and PLR = platelet-to-lymphocyte ratio.

The ROC curves for NLR and PLR were created to determine whether the baseline of these biomarkers was predictive of amputation, mortality, and the composite endpoint of amputation and mortality in all patients (Figure 2). The optimal cut-off obtained from Youden’s index, areas under the curve (AUC), and the predictive accuracy of the ratios are listed in Table 3.

Depending on the optimal cut-off value according to the ROC, the outcomes were further analyzed after dividing the patients into paired groups: low NLR/ high NLR and low PLR/high PLR. In all high NLR and high PLR groups, there was a higher incidence of all adverse outcomes, as seen in Table 4. Furthermore, the RC stratification analyzed the outcomes according to the optimal cut-off values of NLR and PLR. There were statistically higher numbers of amputations, deaths, and both (composite endpoint) in all high ratio groups, with the exception of the PLR concerning the composite endpoint in the RC IIA (*p* = 0.056) and the PLR concerning all outcomes in the RC III (*p* = 0.057, *p* = 0.07, and *p* = 0.07) Table 4).

In the ROC analysis, an NLR value higher than 4.33 was strongly associated with all the studied outcomes. Also, a PLR > 143.34 was associated with all the studied adverse outcomes, except the composite endpoint for all RC grades (Supplementary Figures S1 and S2).

A multivariate analysis showed that a high baseline value for NLR and PLR was an independent predictor of adverse outcomes for all recruited patients. Furthermore, for all hospitalized patients, the RC grade III was an independent predictor of a poor prognosis. However, a diagnosis of RC grade IIA acted as a protective factor against any negative events during the study period (Table 5).

## 4. Discussion

This study included 210 individuals diagnosed with ALI with RC grade > I. For all patients, we determined the preoperative values for NLR and PLR and monitored 30-day outcomes for the amputation rate, mortality rate, and a composite endpoint of amputation and mortality. The most importing finding of our study is that a high baseline value for NLR and PLR was an independent predictor of amputation (OR:11.09; 95% CI: 5.48–22.42; *p* < 0.0001; and OR:8.97; 95% CI: 4.44–18.16; *p* < 0.0001), mortality (OR:22.24; 95% CI: 9.61–51.47; *p* < 0.0001; and OR:8.32; 95% CI: 3.90–17.73; *p* < 0.0001), and composite endpoint (OR:21.93; 95% CI: 7.91–60.79; *p* < 0.0001; and OR:9.98; 95% CI: 3.89–25.55; *p* < 0.0001), respectively. Furthermore, for all hospitalized patients, the RC grade III (OR:7.33; 95% CI: 3.73–14.26; *p* < 0.0001) was an independent predictor of amputation (OR:7.33; 95% CI: 3.73–14.26; *p* < 0.0001), mortality (OR:8.40; 95% CI: 4.08–17.31; *p* < 0.0001), and composite endpoint (OR: 10.70; 95% CI: 4.48–25.56; *p* < 0.0001), respectively.

Currently, ALI is considered an emergency with a high risk for a negative outcome (high rate of amputation and mortality) [1], regardless of the promptness of therapeutic management and the type of treatment chosen be it endovascular, surgical revascularization, or fibrinolysis [30,31].

A high level of inflammation at the time of admission indicates that the patient has advanced atherosclerosis or significant endothelial damage in an acute scenario that triggers an inflammatory response. The involved inflammatory pathways render the patient more prone to developing complications that may culminate with multiple system and organ failure (MSOF). Thus, the associated values of the two biomarkers can help raise clinical awareness of the necessity to actively look for the initial signs of organ insufficiencies.

Elevated NLR and PLR levels in peripheral artery disease have been linked to increased amputation rates, severe cardiovascular events, and death [32,33]. High values of these ratios were also associated with poor outcomes in the evolution of specific diseases, such as abdominal aortic aneurysm (AAA), chronic threatening limb ischemia (CTLI), and other cardiovascular emergencies [13,27,34,35,36,37,38,39,40,41,42,43]. These biomarkers also proved to be predictive in oncology and oncological surgery, particularly in colorectal cancer, pancreatic cancer, gastric cancer, and cardiology and cardiac surgery [9,10,11,12,13,14,15,16,44,45,46].

In a study by Gonzales-Fajardo et al., 561 patients diagnosed with critical limb ischemia were prospectively followed after infrainguinal revascularization [47]. They found that an NLR value > 5 was independently associated with five years of amputation-free survival, with a hazard ratio (HR) of 2.325, 95% CI 1.732–3.121. In a paper published by Tasoglu et al., a value of NLR ≥ 5.2 (83% sensitivity and 63% specificity) was associated with a higher rate of amputation at 30 days in patients who had undergone an embolectomy for ALI [28]. In addition, Spark et al. and Chan et al. showed that an NLR value > 5.25 is an independent predictive factor for all mortality causes (HR 2.3, 95% CI 1.2–4.2; *p* = 0.007) in 149 patients diagnosed with critical limb ischemia (CLI) [48] and 83 patients with CLI after infrapopliteal angioplasty, respectively, (HR 1.97, 95% CI 1.08–3.62; *p* = 0.03) [49].

In a study by Coelho et al., an NLR ≥ 5.4 was associated with a higher incidence of amputation and mortality (90.5% sensitivity and 73.6% specificity) in 345 RC IIA and IIB ALI [50]. Similarly, Pasqui et al. proposed NLR cut-off values of 5.57 and 6.66 as prognostic factors for mortality and amputation, respectively, in 168 RC IIA and IIB ALI [29].

In terms of the PLR, values above 160 have been related to increased amputation rates in patients with CLI, according to a study published by Songur et al. [51]. Lee et al. studied 95 patients postinfrainguinal angioplasty with stent implantation and followed the target vessel restenosis and found that an NLR ≥ 2.75 (87.5% sensitivity and 34.9% specificity) and a PLR ≥ 91 (81.3% sensitivity and 50.8% specificity) were related to an increased incidence of restenosis [52]. Furthermore, high NLR values were correlated with peripheral arterial disease, and high PLR values were associated with the presence of osteomyelitis. In a paper published by Zhou et al., a PLR value > 171 was associated with severe coronary artery stenosis (OR 2.393; 95% CI 1.394–4.108; *p* = 0.002) [53]. Additionally, Gungor et al. highlighted PLR values >119.3 as an independent predictive factor for postoperative AF after coronary artery bypass graft surgery (*p* = 0.005) in 125 patients [54].

In our study, by comparing the high and low values of the ratios for each RC grade, we found significant predictive values for amputation and mortality in both the early and late stages of clinical presentation, with a few exceptions, as follows: for PLR, there was no difference in the amputation and mortality rates of the patients with high RC grades and the composite endpoint for both early and advanced stages based on RC. This can be explained by the poor overall outcome in these advanced stages when patients present with extensive, irreversible soft tissue damages, inflammatory activation, and all cell lines involved (including platelets). However, for early stages of the disease, it is questionable what generates such findings, beyond the chronic evolution of the inflammation process involved in peripheral arterial disease. Of all the ratios, the NLR was found to be the most consistent in predicting an adverse outcome, similar to all other results in the literature.

Risk stratification in ALI patients is crucial in determining the best treatment strategy. Although paraclinical evaluations, laboratory testing, and clinical examinations help forecast probable consequences, the management and prognosis of ALI patients remain a challenge for healthcare providers. Given the significant predictive value of the NLR and PLR and their ease of use in the clinical setting, the values of these markers can be used to help estimate and control probable aggravations related to the Rutherford classification.

Despite the statistically significant results supporting the predictive potential of these ratios, our study has significant limitations that have to be considered. As a single-center study, it included a relatively small number of patients. Another limitation is the short follow-up and the restricted patient management in terms of surgical approach (vs. thrombolysis and endovascular solutions). Considering these limitations, the conclusions might not accurately reflect the experience of other vascular clinics worldwide and cannot be generalized. In the future, a prospective study with long-term outcome monitoring is advised, as well as further research conducted to monitor the relationship between preoperative and postoperative NLR and PLR values and the evolution of ALI patients.

## 5. Conclusions

The NLR and PLR are excellent predictors of risks associated with ALI for primary and secondary prevention. Our study showed that increased preoperative values for NLR and PLR are indicators of poor outcomes in patients with RC grades II and III ALI. Acknowledging these crucial biomarkers will become increasingly important in risk detection and treatment strategies. Due to their simplicity, these markers should be used more frequently in the daily clinical assessment of ischemic vascular crises.

## Figures and Tables

**Table 1:** Demographic data, comorbidities, risk factors, laboratory findings, Rutherford classification, type of surgery, and outcomes for all patients.

Variables	All Patients n = 210
Age mean ± SD (min-max)	69.56 ± 8.21 (51–92)
Male sex no. (%)	153 (72.86%)
Comorbidities and risk factors	
AH, no. (%)	175 (83.33%)
IHD, no. (%)	169 (80.48%)
AF, no. (%)	47 (22.38%)
MI, no. (%)	69 (32.86%)
CHF, no. (%)	127 (60.48%)
COPD, no. (%)	54 (25.71%)
CKD, no. (%)	42 (20%)
T2D, no. (%)	111 (52.86%)
Stroke, no. (%)	57 (27.14%)
CVI, no. (%)	39 (18.57%)
Tobacco, no. (%)	134 (63.81%)
Obesity, no. (%)	71 (33.81%)
Hyperlipidemia, no. (%)	124 (59.05%)
Laboratory findings	
Hemoglobin g/dL median [Q1–Q3]	12.31 [10.46–113.89]
Hematocrit % median [Q1–Q3]	37.31 [32.24–41.98]
Glucose mg/dl median [Q1–Q3]	106 [93–134.75]
Neutrophils × 10^3^/uL median [Q1–Q3]	6.4 [4.89–8.25]
Lymphocytes × 10^3^/uL median [Q1–Q3]	1.98 [1.54–2.69]
Monocyte × 10^3^/uL median [Q1–Q3]	0.61 [0.47–0.79]
PLT × 10^3^/uL median [Q1–Q3]	255.65 [211.1–317.85]
NLR median [Q1–Q3]	3.07 [2.04–4.79]
PLR median [Q1–Q3]	125.73 [94.82–175.48]
Rutherford grade	
IIA, no. (%)	100 (47.62%)
IIB, no. (%)	45 (21.43%)
III, no. (%)	65 (30.95%)
Type of surgery	
Fogarty embolectomy, no. (%)	145 (69.05%)
By-pass, no. (%)	48 (22.86%)
Major amputation, no. (%)	17 (8.1%)
Outcomes	
Amputation, no. (%)	57 (27.14%) (17) *
Death, no. (%)	47 (22.38%)
Amputation and death, no. (%)	33 (15.71%)

AH = arterial hypertension; IHD = ischemic heart disease; AF = atrial fibrillation; MI = myocardial infarction; CHF = chronic heart failure; COPD = chronic obstructive pulmonary disease; CKD = chronic kidney disease; T2D = type 2 diabetes; CVI = chronic venous insufficiency; SD = standard deviation; and PLT = total platelet count; * Number of major amputations performed as mandatory first performed procedure.

**Table 2:** Demographic data, comorbidities, risk factors, laboratory findings Rutherford classification, type of surgery, and outcomes of the two subgroups divided according to poor outcomes.

Variables	Survivor n = 163	Death n = 47	*p*-Value (OR; CI 95%)
Age mean ± SD (min–max)	69.58 ± 7.81 (52–92)	69.48 ± 9.58 (51–89)	0.95 ^#^
Male sex no. (%)	124 (76.07%)	29 (61.70%)	0.052 ^¥^ (1.97; 0.99–3.93)
Comorbidities and risk factors			
AH, no. (%)	134 (82.21%)	41 (87.23%)	0.41 ^¥^ (0.67; 0.26–1.74)
IHD, no. (%)	131 (80.37%)	38 (80.85%)	0.94 ^¥^ (0.96; 0.42–2.20)
AF, no. (%)	33 (20.25%)	14 (29.78%)	0.16 ^¥^ (0.59; 0.28–1.24)
MI, no. (%)	52 (31.9%)	17 (36.1%)	0.58 ^¥^ (0.82; 0.41–1.63)
CHF, no. (%)	100 (61.35%)	27 (57.44%)	0.62 ^¥^ (1.17; 0.60–2.27)
COPD, no. (%)	41 (25.15%)	13 (27.65%)	0.72 ^¥^ (0.87; 0.42–1.82)
CKD, no. (%)	30 (18.4%)	12 (25.53%)	0.16 ^¥^ (0.58; 0.27–1.25)
T2D, no. (%)	88 (53.99%)	23 (48.93%)	0.54 ^¥^ (1.22; 0.63–2.34)
Stroke, no. (%)	41 (25.15%)	16 (34.04%)	0.22 ^¥^ (0.65; 0.32–1.31)
CVI, no. (%)	29 (17.79%)	10 (21.27%)	0.58 ^¥^ (0.80; 0.35–1.79)
Tobacco, no. (%)	103 (63.19%)	31 (65.95%)	0.72 ^¥^ (0.88; 0.44–1.75)
Obesity, no. (%)	54 (33.13%)	17 (36.1%)	0.69 ^¥^ (0.87; 0.44–1.72)
Hyperlipidemia, no. (%)	94 (57.67%)	30 (63.82)	0.44 ^¥^ (0.77; 0.39–1.51)
Laboratory findings			
Hemoglobin g/dL median [Q1–Q3]	12.6 [10.92–14.15]	10.95 [9.74–12.63]	0.0005 ^§^
Hematocrit % median [Q1–Q3]	38.99 [33.18–42.6]	32.9 [29.95–37.36]	<0.0001 ^§^
Glucose mg/dL median [Q1–Q3]	104 [91.5–132.4]	118 [100.5–140.25]	0.01 ^§^
Neutrophils × 10^3^/uL median [Q1–Q3]	5.83 [4.59–7.57]	8.29 [6.76–10.99]	<0.0001 ^§^
Lymphocytes × 10^3^/uL median [Q1–Q3]	2.13 [1.71–2.90]	1.55 [1.16–1.95]	<0.0001 ^§^
Monocyte × 10^3^/uL median [Q1–Q3]	0.61 [0.46–0.78]	0.68 [0.52–0.98]	0.03 ^§^
PLT × 10^3^/uL median [Q1–Q3]	251.1 [204.25–309.75]	277 [229.2–386.6]	0.02 ^§^
NLR median [Q1–Q3]	0.28 [0.20–0.34]	0.47 [0.33–0.62]	<0.0001 ^§^
PLR median [Q1–Q3]	116.31 [89.03–155.08]	190.54 [145.47–241.38]	<0.0001 ^§^
Rutherford grade			
IIA, no. (%).	93 (57.05%)	7 (14.89%)	<0.0001 ^¥^ (7.59; 3.20–17.95)
IIB, no. (%)	37 (22.69%)	8 (17.02%)	0.40 ^¥^ (1.43; 0.61–3.33)
III, no. (%)	33 (20.24%)	32 (68.08%)	<0.0001 ^¥^ (0.11; 0.05–0.24)
Type of surgery			
Fogarty embolectomy, no. (%)	118 (72.39%)	27 (57.45%)	0.053 ^¥^ (1.94; 0.99–3.80)
By-pass, no. (%)	38 (23.31%)	10 (21.28%)	0.76 ^¥^ (1.12; 0.51–2.47)
Major amputation, no. (%)	7 (4.29%)	10 (21.28%)	0.0006 ^¥^ (0.16; 0.05–0.46)
Outcomes			
Amputation, no. (%)	24 (14.72%) (7) *	33 (70.21%) (10) *	<0.0001 ^¥^ (0.07; 0.03–0.15)

AH = arterial hypertension; IHD = ischemic heart disease; AF = atrial fibrillation; MI = myocardial infarction; CHF = chronic heart failure; COPD = chronic obstructive pulmonary disease; CKD = chronic kidney disease; T2D = type 2 diabetes; CVI = chronic venous insufficiency; SD = standard deviation; PLT = total platelet count; ^#^, Student’s *t*-test; ^¥^, Chi-square test; ^§^, Mann–Whitney test; and ^¥^, Chi-square test; * Numbers in parentheses represent the number of major amputations performed as mandatory first performed procedure.

**Table 3:** ROC curves, optimal cut-off value, AUC, and predictive accuracy of inflammatory markers NLR and PLR.

Variables	Cut-Off	AUC	Std. Error	95% CI	Sensitivity	Specificity	*p*-Value
Amputation in all patients							
NLR	4.23	0.829	0.030	0.769–0.888	70.2%	83%	<0.0001
PLR	136.68	0.785	0.036	0.713–0.856	80.7%	71.2%	<0.0001
Mortality in all patients							
NLR	4.33	0.856	0.029	0.799–0.913	80.9%	84%	<0.0001
PLR	143.34	0.765	0.040	0.686–0.844	76.6%	71.8%	<0.0001
Amputation and mortality in all patients							
NLR	4.33	0.858	0.028	0.802–0.914	84.8%	79.7%	<0.0001
PLR	143.34	0.759	0.043	0.675–0.844	81.8%	68.9%	<0.0001

NLR = neutrophil-to-lymphocyte ratio; MLR = monocyte-to-lymphocyte ratio; PLR = platelet-to-lymphocyte ratio; OR = odds ratio; and CI = confidence interval.

**Table 4:** Univariate analysis of NLR, MLR, PLR, and all adverse event occurrences during the study period for all patients.

	Amputation	Mortality	Amputation and Mortality
low NLR vs. high NLR All patients	18/146 (12.33%) vs. 39/64 (60.94%) *p* < 0.0001 OR:0.09 CI: (0.04–0.18)	9/146 (6.16%) vs. 38/64 (59.37%) *p* < 0.0001 OR:0.04 CI: (0.01–0.10)	5/146 (3.42%) vs. 28/64 (43.75%) *p* < 0.0001 OR:0.04 CI: (0.01–0.12)
Low NLR vs. high NLR RC IIA patients	2/76 (2.63%) vs. 10/24 (41.67%) *p* = 0.0001 OR:0.03 CI: (0.007–0.19)	0/76 (0%) vs. 7/24 (29.17%) *p* = 0.004 OR:0.01 CI: (0.0008–0.27)	0/76 (0%) vs. 4/24 (16.67%) *p* = 0.02 OR:0.02 CI: (0.001–0.57)
Low NLR vs. high NLR RC IIB patients	4/36 (11.11%) vs. 5/9 (55.56%) *p* = 0.007 OR:0.10 CI: (0.01–0.53)	2/36 (5.56%) vs. 6/9 (66.67%) *p* = 0.0005 OR:0.02 CI: (0.004–0.21)	1/36 (2.78%) vs. 3/9 (33.34%) *p* = 0.04 OR:0.1 CI: (0.01–0.99)
Low NLR vs. high NLR RC III patients	12/34 (35.29%) vs. 24/31 (77.42%) *p* = 0.001 OR:0.15 CI: (0.05–0.47)	7/34 (20.59%) vs. 25/31 (80.65%) *p* < 0.0001 OR:0.06 CI: (0.01–0.21)	4/34 (11.76%) vs. 21/31 (67.74%) *p* = 0.02 OR:0.05 CI: (0.005–0.64)
	**Amputation**	**Mortality**	**Amputation and Mortality**
Low PLR vs. high PLR All patients	14/128 (10.94%) vs. 43/82 (52.44%) *p* < 0.0001 OR:0.11 CI: (0.05–0.22)	11/128 (8.59%) vs. 36/82 (43.9%) *p* < 0.0001 OR:0.12 CI: (0.05–0.25)	6/128 (4.69%) vs. 27/82 (32.93%) *p* < 0.0001 OR:0.10 CI: (0.03–0.25)
Low PLR vs. high PLR RC IIA patients	4/74 (5.41%) vs. 8/26 (30.77%) *p* = 0.002 OR:0.12 CI: (0.03–0.47)	2/74 (2.7%) vs. 5/26 (19.23%) *p* = 0.01 OR:0.11 CI: (0.02–0.64)	1/74 (1.35%) vs. 3/26 (11.54%) *p* = 0.056 OR:0.10 CI: (0.01–1.05)
Low PLR vs. high PLR RC IIB patients	3/35 (8.57%) vs. 6/10 (60%) *p* = 0.001 OR:0.06 CI: (0.01–0.35)	3/35 (8.57%) vs. 5/10 (50%) *p* = 0.006 OR:0.09 CI: (0.01–0.52)	1/35 (2.86%) vs. 3/10 (30%) *p* = 0.02 OR:0.06 CI: (0.006–0.76)
Low PLR vs. high PLR RC III patients	7/19 (36.84%) vs. 29/46 (63.04%) *p* = 0.057 OR:0.34 CI: (0.11–1.03)	6/19 (31.58%) vs. 26/46 (56.52%) *p* = 0.07 OR:0.35 CI: (0.11–1.09)	4/19 (21.05%) vs. 21/46 (45.65%) *p* = 0.07 OR:0.31 CI: (0.09–1.10)

NLR = neutrophil-to-lymphocyte ratio; PLR = platelet-to-lymphocyte ratio; OR = odds ratio; and CI = confidence interval.

**Table 5:** Multivariate analysis on new adverse event occurrence during the entire study period.

Variables	Amputation			Mortality			Amputation and Mortality		
	OR	95% CI	*p*-Value	OR	95% CI	*p*-Value	OR	95% CI	*p*-Value
AF	1.35	0.66–2.73	0.405	1.67	0.80–3.47	0.169	1.95	0.87–4.40	0.104
MI	1.27	0.67–2.41	0.454	1.21	0.61–2.38	0.583	1.02	0.46–2.25	0.949
Stroke	1.34	0.69–2.62	0.378	1.53	0.76–3.09	0.229	1.42	0.64–3.16	0.385
Tobacco	1.65	0.85–3.20	0.137	1.12	0.57–2.23	0.728	1.16	0.52–2.54	0.710
RC IIA	0.197	0.09–0.40	<0.001	0.13	0.05–0.31	<0.001	0.11	0.03–0.34	<0.001
RC IIB	0.609	0.27–1.36	0.227	0.69	0.30–1.62	0.405	0.45	0.15–1.37	0.164
RC III	7.33	3.73–14.26	<0.001	8.40	4.08–17.31	<0.001	10.70	4.48–25.56	<0.001
High NLR	11.09	5.48–22.42	<0.001	22.24	9.61–51.47	<0.001	21.93	7.91–60.79	<0.001
High PLR	8.97	4.44–18.16	<0.001	8.32	3.90–17.73	<0.001	9.98	3.89–25.55	<0.001

AF = atrial fibrillation; MI = myocardial infarction; NLR = neutrophil-to-lymphocyte ratio; PLR = platelet-to-lymphocyte ratio; RC = Rutherford classification; OR = odds ratio; and CI = confidence interval.

## Supplementary Materials

The following supporting information can be downloaded at: https://www.mdpi.com/article/10.3390/life12060822/s1, Figure S1: ROC curve analysis: (**A**) for NLR concerning amputation rate in RC IIA patients (AUC: 0.887; *p* < 0.0001), (**B**) for NLR concerning mortality rate in RC IIA patients (AUC: 0.917; *p* < 0.0001), (**C**) for NLR concerning amputation and mortality rate in IIA patients (AUC: 0.940; *p* = 0.003), (**D**) for NLR concerning amputation rate in RC IIB patients (AUC: 0.843; *p* = 0.002), (**E**) for NLR concerning mortality rate in RC IIB patients (AUC: 0.878; *p* = 0.001), (**F**) for NLR concerning amputation and mortality rate in IIB patients (AUC: 0.848; *p* = 0.023), (**G**) for NLR concerning amputation rate in RC III patients (AUC: 0.695; *p* = 0.007), (**H**) for NLR concerning mortality rate in RC III patients (AUC: 0.772; *p* < 0.0001), and (**I**) for NLR concerning amputation and mortality rate in III patients (AUC: 0.772; *p* < 0.0001). Figure S2: ROC curve analysis: (**A**) for PLR concerning amputation rate in RC IIA patients (AUC: 0.787; *p* = 0.001), (**B**) for PLR concerning mortality rate in RC IIA patients (AUC: 0.805; *p* = 0.007), and (**C**) for PLR concerning amputation and mortality rate in RC IIA patients (AUC: 0.784; *p* = 0.055), (**D**) for PLR concerning amputation rate in RC IIB patients (AUC: 0.725; *p* = 0.038), (**E**) for PLR concerning mortality rate in RC IIB patients (AUC: 0.780; *p* = 0.014), (**F**) for PLR concerning amputation and mortality rate in RC IIB patients (AUC: 0.726; *p* = 0.14), (**G**) for PLR concerning amputation rate in RC III patients (AUC: 0.684; *p* = 0.011), (**H**), for PLR concerning mortality rate in RC III patients (AUC: 0.653; *p* = 0.03), and (**I**) for PLR concerning amputation and mortality rate in RC III patients (AUC: 0.616; *p* = 0.118).
