# Supplementary figures and images for: Neutrophil-to-Lymphocyte Ratio and Platelet-to-Lymphocyte Ratio Impact on Predicting Outcomes in Patients with Acute Limb Ischemia
Source: Life (Basel). 2022 May 31;12(6):822. doi: 10.3390/life12060822 (PMC9225565; doi:10.3390/life12060822)

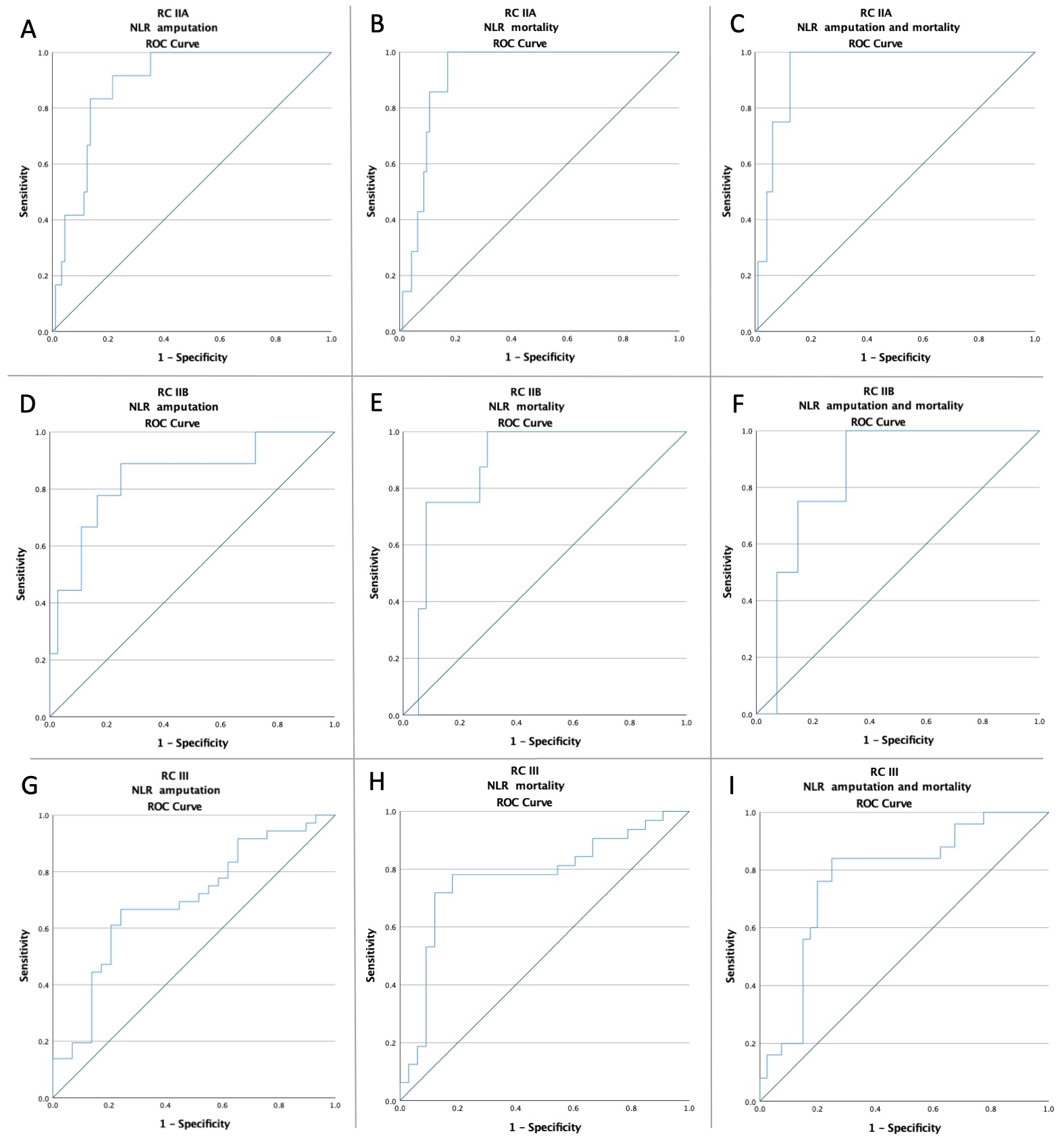

Supplement: Supplementary file 1 [file life-12-00822-s001.zip › Supplementary Figure S1.tiff]

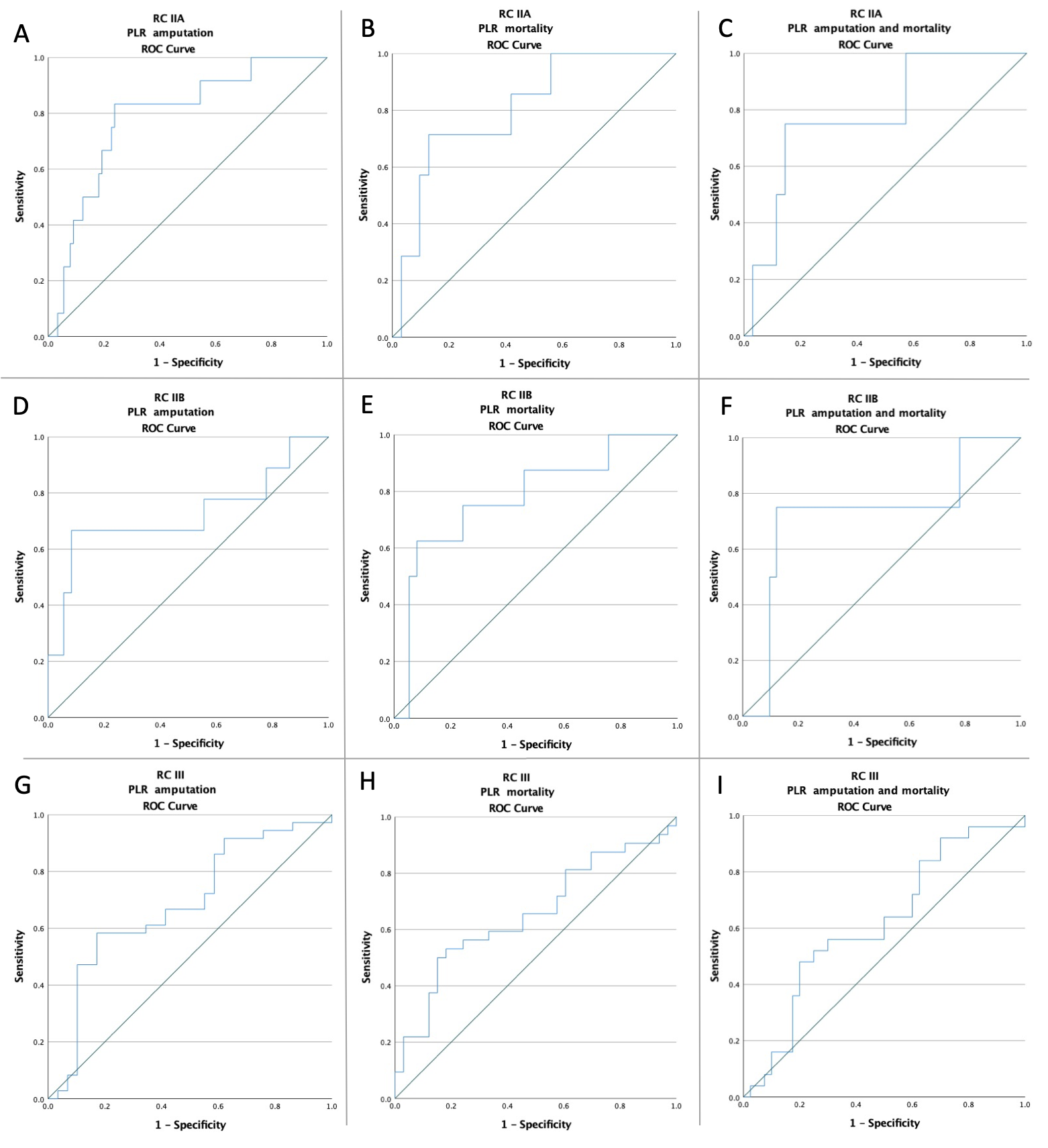

Supplement: Supplementary file 1 [file life-12-00822-s001.zip › Supplementary Figure S2.tiff]
